# The impact of squamous cell carcinoma histology on outcomes in nonmetastatic pancreatic cancer

**DOI:** 10.1002/cam4.2851

**Published:** 2020-01-16

**Authors:** Joshua D. Gruhl, Ignacio Garrido‐Laguna, Samual R. Francis, Kajsa Affolter, Randa Tao, Shane Lloyd

**Affiliations:** ^1^ Department of Radiation Oncology Huntsman Cancer Hospital University of Utah Salt Lake City UT USA; ^2^ Department of Internal Medicine Huntsman Cancer Hospital University of Utah Salt Lake City UT USA; ^3^ Department of Anatomic Pathology Huntsman Cancer Hospital University of Utah Salt Lake City UT USA

**Keywords:** ductal adenocarcinoma, nonmetastatic, outcomes analysis, pancreatic cancer, squamous cell carcinoma pathology

## Abstract

**Background:**

The prognosis for nonmetastatic, primary pancreatic squamous cell carcinoma (SCC) is thought to be poor compared with adenocarcinoma (AC); however, this is based on limited data. Additionally, the optimal definitive treatment strategy for nonmetastatic pancreatic SCC is unknown.

**Methods:**

We analyzed patients with nonmetastatic pancreatic cancer using the National Cancer Database for patients diagnosed from 2006 to 2014. Patients were analyzed according to histology—only AC, adenosquamous carcinoma (A‐SCC), and SCC were included. The primary endpoint was overall survival (OS) from the time of diagnosis.

**Results:**

A total of 94 928 cases were included; 94 016 AC, 757 A‐SCC, and 155 SCC. Median OS was lower for SCC (8.67 months), compared to AC (13.93 months) and A‐SCC (12.71 months, *P *< .001). SCC was resected less often (25.5% vs 46.7% and 74.5%). On subgroup analysis of patients with pancreatic SCC, factors on multivariate analysis associated with improved survival included surgery (HR 0.19, *P *< .001), and chemotherapy (HR 0.22, *P* = .01). In 38 patients with SCC undergoing surgical resection, median OS improved (MS = 6.8 months without surgery vs 21.3 months with surgery, *P *< .001).

**Conclusions:**

Nonmetastatic pancreatic SCC presents with more advanced disease, which is less often surgically resected or treated with any definitive local therapy. In contrast, AC and A‐SCC behave more similarly and have higher surgical resection rates and improved survival. In patients with nonmetastatic SCC of the pancreas, surgical resection provides the most significant survival benefit, with systemic chemotherapy providing a less significant benefit, and localized radiation providing no statistical benefit for any subgroup.

## Graphical abstract

Nonmetastatic, primary pancreatic squamous cell carcinoma (SCC) presents with more aggressive clinicopathologic disease, which is less often surgically resected or treated with any definitive local therapy, compared to pancreatic adenocarcinoma and adenosquamous carcinoma. In patients with non‐metastatic SCC of the pancreas, surgical resection provides the most significant survival benefit, with chemotherapy providing a less significant benefit.

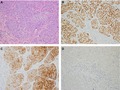

## 1 INTRODUCTION

**Figure 1:**
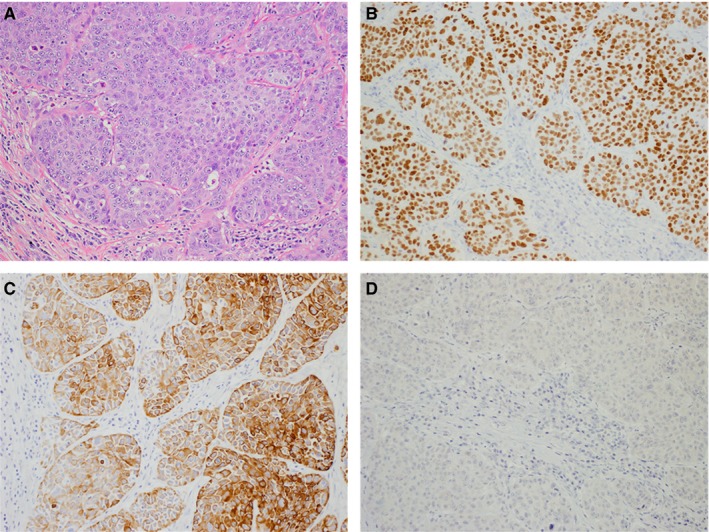
Histology and select immunohistochemistry for a pure pancreatic squamous cell carcinoma (20× objective); (A) Hematoxylin and Eosin routine stain, (B) p63 diffuse nuclear positivity, (C) Cytokeratin 5/6 diffuse cytoplasmic and membranous positivity, (D) Synaptophysin negative

The normal pancreas consists of bicarbonate‐secreting ductal cells, acinar cells, centroacinar cells, stellate cells, and hormone‐secreting endocrine islet cells.^1^ Ductal adenocarcinomas (AC) represent greater than 85% of pancreatic neoplasms; less common histologies include neuroendocrine, acinar, pancreatoblastomas, and solid‐pseudopapillary neoplasms, among others.^1^, ^2^ Even rarer are adenosquamous carcinoma (A‐SCC) and squamous cell carcinoma (SCC). The normal pancreas is devoid of squamous cells; however, squamous metaplasia is often detected in pancreatic autopsy specimens. In a study of 206 pancreas autopsy specimens from patients with no known history of cancer, Mukada et al reported squamous metaplasia in 16.4% of cases.^3^ It is unclear how primary SCC develops in an organ histologically devoid of native squamous cells; theories include development from a progenitor cell, malignant transformation of inflammatory‐induced squamous metaplasia (possibly related to chronic pancreatitis or pancreatic stent placement), and squamous transformation of AC.^4^, ^5^ (Figure 1) Others hypothesize that primary SCC of the pancreas is, in fact, metastatic SCC arising in an occult primary elsewhere in the body.^6^

Data on primary pancreatic SCC and A‐SCC are sparse, as they represent only 0.5%‐2%, and 5%, of pancreatic neoplasms, respectively.^7^, ^8^ Incidence rates for primary pancreatic SCC and A‐SCC are increasing.^9^ The prognosis for primary pancreatic SCC is thought to be poor compared with AC; however, this is based primarily on case reports and small case series.^9^, ^10^, ^11^, ^12^, ^13^ Treatment paradigms for nonmetastatic pancreatic AC are well established; however, in cases of nonmetastatic, primary SCC, and A‐SCC of the pancreas, the optimal definitive treatment strategy is unknown. No large studies exist to help guide clinical treatment decisions. Additionally, the National Comprehensive Cancer Network^14^ pancreatic cancer guidelines only comment on AC.^14^

We hypothesize that nonmetastatic SCC of the pancreas presents with more aggressive clinical and pathologic features, and results in worse overall survival (OS), compared with AC and A‐SCC. We sought to determine if nonmetastatic, primary pancreatic SCC patients receive definitive local treatment less often than do those with AC or A‐SCC. We also investigated whether surgical resection and chemotherapy are independently associated with improved OS in nonmetastatic primary SCC and A‐SCC. Using the National Cancer Database (NCDB), which pulls data from over 1500 accredited cancer facilities and includes over 70% of newly diagnosed cancer cases in the United States, we analyzed patients with nonmetastatic pancreatic cancer and sought to determine variations in outcomes between AC, A‐SCC, and SCC. Additionally, to help guide future treatment recommendations, we sought to identify prognostic factors for OS in primary, nonmetastatic pancreatic SCC—specifically, the impact of surgical resection, chemotherapy, and localized radiotherapy.

## 2 METHODS AND MATERIALS

### 2.1 NCDB analysis

We analyzed patients with nonmetastatic pancreatic cancer using the NCDB for patients diagnosed from 2006 to 2014. All cases of nonmetastatic pancreatic cancer were queried, but only patients with pathologic confirmation of AC, A‐SCC, or SCC were included for analysis. Patients were excluded for the following: clinical stage 0, metastatic disease, unknown histology, <3 months follow‐up or death within 3 months, or unknown follow‐up months. When analyzing receipt of radiation by histologic subtype, only patients treated with radiation with definitive intent were included—this required that radiation was delivered to the primary site (pancreas) and doses between 36 and 60 Gy were used. Patient demographics, clinical characteristics, and treatment details used for analysis included: age at diagnosis, sex, Charlson comorbidity score, resectability, tumor grade, lymphovascular space invasion (LVSI), CA 19‐9, clinical T‐stage, clinical N‐stage, pathologic T‐stage, pathologic N‐stage, margin status, surgery received, type of surgery, radiation received, radiation modality, chemotherapy received, chemotherapy type (single‐ or multi‐agent), and sequencing of chemotherapy.

The primary endpoint was OS based on time of diagnosis to the date of death. Patients were censored at the date of last follow‐up. Chi‐squared analysis and multivariate logistic regression were used to compare demographics, clinical characteristics, and treatment details between the AC and SCC groups.

Kaplan‐Meier, and univariate (UVA) and multivariate (MVA) Cox proportional hazards modeling were used to analyze factors associated with OS. Variables that had an association with survival, defined as *P* < .2, on UVA were included in MVA***.*** Statistical analyses were performed in STATA 14.2 (StataCorp). A *P* < .05 was considered statistically significant. All tests were two sided. The study was exempt from the institutional review board.

### 2.2 Literature review

Eligible studies were retrieved in PubMed with the end of search date January 3, 2019, using the following search terms: (pancreas or pancreatic) AND cancer AND squamous. Eligible articles included retrospective and prospective studies, as well as case series and case reports. Articles referring exclusively to A‐SCC were excluded from the present review.

## 3 RESULTS

A total of 114 104 cases of nonmetastatic pancreatic cancer diagnosed from 2006 to 2014 were retrieved from the NCDB; this included all histological subtypes. As illustrated in Table S2, a majority of the cases (82.4%) were AC, followed by neuroendocrine tumor (6.2%), carcinoma NOS (5.09%), and malignant neoplasm/tumor (3.54%). After selecting only for AC, A‐SCC, and SCC, 94 928 patients remained for analysis; 94 016 in the AC group, 757 in the A‐SCC group, and 155 in the SCC group. Patient demographics and tumor characteristics are summarized in Table 1. Mean age at diagnosis was 67.7 years for the AC group, 67.1 years for the A‐SCC group, and 68.5 years for the SCC group. There was no difference in age at diagnosis, male:female ratio, or Charlson comorbidity score between AC and SCC.

**Table 1:** Demographics and clinical characteristics by histologic subtype

	Adenocarcinoma (AC)	Adenosquamous carcinoma (A‐SCC)	Squamous cell carcinoma (SCC)	Total: 94 928
	N = 94 016	N = 757	N = 155	
	N (%)	N (%)	N (%)	*P* (for AC vs SCC)
Age				
≤50	6951 (7.0)	56 (7.0)	11 (7.0)	.89
>50	87 065 (93.0)	701 (93.0)	144 (93.0)	
Sex				
Male	46 280 (49.2)	381 (50.3)	79 (51.0)	.67
Female	47 736 (50.8)	376 (49.7)	76 (49.0)	
Charlson comorbidity score				
0	64 984 (69.1)	518 (68.4)	101 (65.2)	.55
1	22 708 (24.2)	186 (25.6)	43 (27.7)	
2+	6324 (6.7)	53 (7.0)	11 (7.1)	
Tumor location				
Head	63 736 (67.8)	385 (50.9)	75 (48.4)	**<.001**
Body	9428 (10.0)	97 (12.8)	34 (21.9)	
Tail	6035 (6.4)	162 (21.4)	20 (12.9)	
Pancreatic duct	644 (0.7)	4 (0.5)	0 (0.0)	
Other	14 173 (15.1)	109 (14.4)	26 (16.8)	
Tumor grade				
Low	31 404 (62.5)	190 (33.7)	18 (21.7)	**<.001**
High	18 807 (37.5)	374 (66.3)	65 (78.3)	
LVSI				
Negative	11 452 (12.2)	126 (16.6)	15 (9.7)	**.02**
Positive	8559 (9.1)	142 (18.8)	5 (3.2)	
Unknown	74 005 (78.7)	489 (64.6)	135 (87.1)	
CA 19‐9 (U/mL)				
≤35	8860 (9.4)	87 (11.5)	18 (11.6)	.15
35‐90	8341 (8.9)	75 (9.9)	18 (11.6)	
>90	15 631 (16.6)	111 (14.7)	19 (12.3)	
Unknown	61 184 (65.1)	484 (63.9)	100 (64.5)	
Clinical T‐stage				
cT0	160 (0.2)	2 (0.4)	0 (0.0)	.15
cT1	5896 (8.4)	24 (4.3)	7 (5.7)	
cT2	17 414 (24.8)	168 (30.3)	21 (17.1)	
cT3	27 424 (39.0)	253 (45.8)	53 (43.1)	
cT4	19 392 (27.6)	106 (19.2)	42 (34.2)	
Clinical N‐stage				
cN0	47 654 (68.8)	373 (69.2)	74 (66.1)	.38
cN1	21 621 (31.2)	166 (30.8)	38 (33.9)	
Clinical stage group				
I	17 966 (26.4)	154 (28.9)	20 (17.1)	**.03**
II	30 820 (45.3)	271 (50.8)	53 (45.3)	
III	19 304 (28.4)	108 (20.3)	44 (37.6)	
Resectability				
Resectable/borderline resectable	72 042 (79.9)	641 (85.9)	109 (75.7)	.11
Unresectable	18 120 (20.1)	105 (14.1)	37 (25.3)	
Pathologic T‐stage				
pT0	159 (0.4)	3 (0.5)	1 (2.9)	**.03**
pT1	3272 (7.8)	7 (1.3)	2 (5.9)	
pT2	7239 (17.1)	103 (18.7)	8 (23.5)	
pT3	29 082 (68.9)	406 (73.6)	18 (52.9)	
pT4	2468 (5.8)	33 (6.0)	5 (14.7)	
pTis	8 (0.0)	0 (0.0)	0 (0.0)	
Pathologic N‐stage				
pN0	16 209 (38.7)	227 (42.0)	16 (51.6)	.14
pN1	25 665 (61.3)	314 (58.0)	15 (49.4)	
Margin status				
Negative	31 584 (84.0)	431 (86.7)	26 (81.3)	.68
Positive	6028 (16.0)	66 (13.3)	6 (18.8)	

Note

Data presented as N (%) unless otherwise noted. *P* values given by Pearson's chi‐squared and two‐sided *t* tests for categorical and continuous variables, respectively. Low‐grade category included well‐differentiated and moderately differentiated tumors; high‐grade category included poorly differentiated tumors.

The bolded values are statistically significant to *P* < .05.

Abbreviations: LVSI indicates lymphovascular space invasion; N, numbers; N‐stage, clinical nodal stage as per American Joint Committee on Cancer 7th Edition; T‐stage, clinical tumor stage as per American Joint Committee on Cancer 7th Edition.

All three histologies were found most often in the pancreatic head (67.8% for AC, 50.9% for A‐SCC, and 48.4% for SCC, *P *< .001 for AC vs SCC); SCC was found in the pancreatic body in 21.9% of SCC cases, nearly double the rate for AC and A‐SCC (10.03% and 12.81%, respectively). SCC tumors were more often poorly differentiated, rather than well or moderately differentiated (78.3% vs 21.7%, *P *< .001) compared with AC (62.5% vs 37.5%). SCC was more likely to present as stage group III disease, compared with AC (37.6% vs 28.4%, *P* = .03) (Table 1).

A multivariate logistic regression analysis (MVA) for patient demographics and tumor characteristics between AC and SCC histology is summarized in Table S1. On MVA, factors predictive of SCC histology included tumor location in the body of the pancreas (OR 4.14, *P* = .02), tumor location in the tail of the pancreas (OR 4.20, *P* = .01), and high‐grade (OR 10.48, *P* < .01). The following factors were not predictive for SCC histology on MVA: Age, Sex, Charlson Comorbidity Score, LVSI, Clinical Stage Group, and Resectability (Table S1).

**Figure 2:**
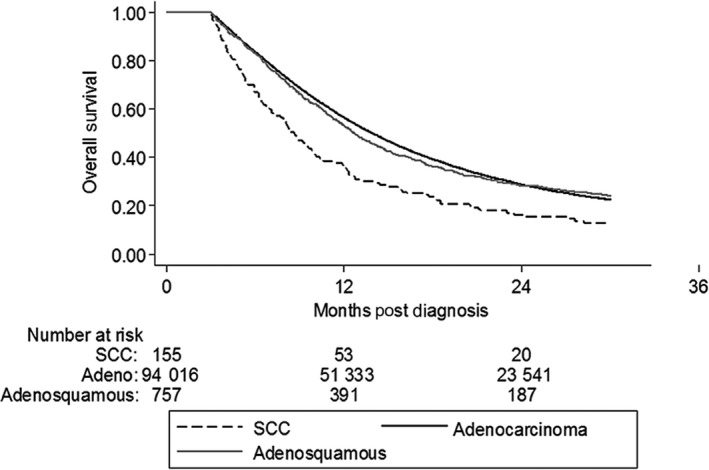
Kaplan‐Meier analysis of overall survival for nonmetastatic pancreatic cancer by histologic subtype. Adeno, adenocarcinoma; SCC, squamous cell carcinoma

**Figure 3:**
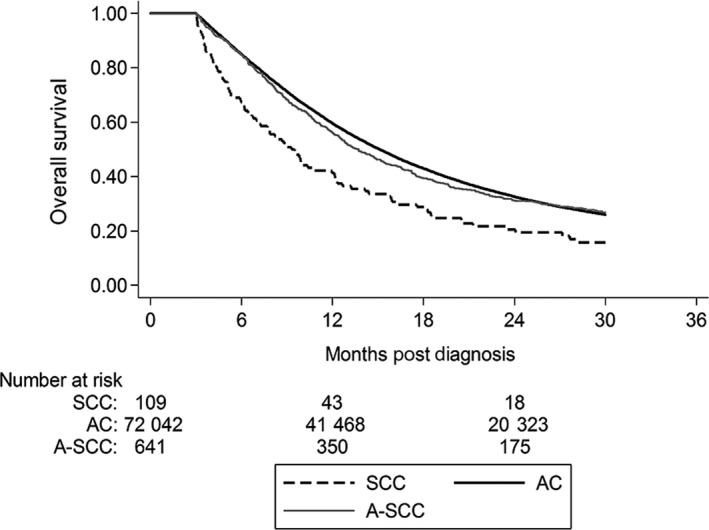
Kaplan‐Meier analysis of overall survival for nonmetastatic, resectable, and borderline resectable pancreatic cancer by histologic subtype. AC, adenocarcinoma; A‐SCC, adenosquamous carcinoma; SCC, squamous cell carcinoma

Median follow‐up was 13.24 months. Median OS was lower for SCC (8.67 months, 95% CI: 7.23‐9.92 months), compared to AC (13.93 months, 95% CI: 13.83‐14.03 months), and A‐SCC (12.71 months, 95% CI: 11.89‐13.73 months, *P *< .001) (Table 2). On Kaplan‐Meir analysis, AC had the highest OS, followed by A‐SCC. SCC had the lowest OS (Figure 2). This was true for patients with resectable/borderline resectable disease (Figure 3), as well as for those with unresectable disease (Figure S1).

**Table 2:** Survival in nonmetastatic pancreatic cancer by histologic type

Histology	6‐mo OS (%)	12‐mo OS (%)	18‐mo OS (%)	24‐mo OS (%)	30‐mo OS (%)	Median OS
Adenocarcinoma (AC)	83.7	56.5	39.3	28.9	22.5	14.0 mo
Adenosquamous carcinoma (A‐SCC)	83.0	53.0	36.2	28.4	24.2	12.7 mo
Squamous cell carcinoma (SCC)	67.1	36.2	23.8	16.3	12.9	8.67 mo

Abbreviation: OS, overall survival.

Table 3 summarizes local treatment received by the histologic subtype. Patients with SCC were significantly less likely to undergo surgical resection compared to patients with AC (25.5% vs 46.7%, *P *< .001). Regardless of histology, most patients did not receive localized radiotherapy to the pancreas (30.5% for AC and 25.4% for SCC). There was no difference in utilization rates of chemotherapy between AC and SCC (66.7% vs 62.5%, *P* = .28) (Table 3). On MVA, neither treatment with surgery, nor treatment with radiation, was predictive of SCC histology (Table S1).

**Table 3:** Local treatment(s) received, stratified by the histologic group

	Adenocarcinoma (AC)	Adenosquamous carcinoma (A‐SCC)	Squamous cell carcinoma (SCC)	Total: 94 928
	N = 94 016	N = 757	N = 155	
	N (%)	N (%)	N (%)	*P* (for AC vs SCC only)
Treated with surgery				
No	48 615 (53.32)	187 (25.10)	111 (74.50)	**<.001**
Yes	42 564 (46.68)	558 (74.90)	38 (25.50)	
Surgery type				
Pancreatectomy	41 607 (97.75)	552 (98.92)	32 (84.21)	**<.001**
Local excision	957 (2.25)	6 (1.08)	6 (15.79)	
Treated with radiation (definitive)				
No	56 481 (69.45)	492 (74.55)	97 (74.62)	.35
Yes	24 839 (30.54)	168 (25.45)	33 (25.38)	
Treated with chemotherapy				
No	29 835 (33.27)	223 (31.19)	54 (37.50)	.28
Yes	59 834 (66.73)	492 (68.81)	90 (62.50)	
Chemotherapy type				
Single agent chemo	33 050 (60.72)	286 (62.72)	38 (47.50)	**.02**
Multiagent chemo	21 384 (39.28)	170 (37.28)	42 (52.50)	
Timing of chemotherapy if receiving surgery + chemotherapy				
Neoadjuvant	3269 (12.89)	30 (9.26)	2 (10.53)	.54
Adjuvant	20 723 (81.74)	288 (88.89)	17 (89.47)	
Neoadjuvant + adjuvant	1359 (5.36)	6 (1.85)	0 (0.0)	
Treatment modality: CHT/RT				
No CHT or RT	27 522 (31.13)	204 (28.81)	47 (33.81)	.38
RT alone	2142 (2.42)	18 (2.54)	6 (4.32)	
CHT alone	25 643 (29.01)	253 (35.73)	40 (28.78)	
CHT + RT	33 094 (37.44)	233 (32.91)	46 (33.09)	

Note

Data presented as N (%) unless otherwise noted. *P* values given by Pearson's chi‐squared and two‐sided *t* tests for categorical and continuous variables, respectively. Patients classified as treated with surgery included those receiving pancreatectomy or local excision. To be classified as receiving definitive radiation, doses >45 Gy were required.

The bolded values are statistically significant to *P* < .05.

Abbreviations: CHT, chemotherapy; RT, radiotherapy.

On subgroup analysis of nonmetastatic, pure primary pancreatic SCC, we found on Cox UVA that surgery (HR 0.22, *P* < .001) was significantly associated with improved survival (Table S3). In contrast, factors associated with a survival decrement included: high‐grade tumors (HR 2.54, *P* < .01), clinical T3 disease (HR 3.18, *P* = .05), and clinical T4 disease (HR 4.05, *P* = .02). On Cox MVA, surgery (HR 0.19, *P* < .001) and receipt of chemotherapy (HR 0.22, *P* = .01) were associated with improved OS (Table S3); cT2 disease (HR 8.88, *P* = .01), cT3 disease (HR 15.13, *P* < .001), cT4 disease (HR 6.03, *P* = .05) were associated with worse OS. There was no significant difference in OS on MVA between those SCC patients receiving radiation and those not receiving radiation. In the 38 patients with SCC undergoing surgical resection, median OS (MS) was improved (for patients not undergoing surgery, MS = 6.8 months vs 21.3 months for patients undergoing surgery, *P* < .001).

A PubMed literature search returned six relevant studies of primary SCC of the pancreas (Table S4). The largest of these studies was a SEER analysis of 214 patients; the smallest study included six patients. Median survival ranged from 2.5 to 9 months. Five studies reported the frequency of metastatic disease at presentation; in four of these, >50% of patients had upfront metastatic disease. There were no studies that focused exclusively on nonmetastatic SCC of the pancreas.

## 4 DISCUSSION

Our study is the first large‐scale aggregate database analysis of patients with nonmetastatic, primary SCC of the pancreas. Our analysis found that some aggressive features are more common at diagnosis in SCC compared to AC, including higher grade tumors and more locally advanced disease. Overall, patients with SCC had inferior survival compared to patients with AC and A‐SCC, which have similar survival to one another. For patients with nonmetastatic, primary SCC of the pancreas, surgical resection, and chemotherapy independently improved OS.

The majority of data on pancreatic SCC come from individual case reports and small case series. Poor outcomes are nearly universal, with survival ranging from 1 to 11 months.^7^, ^12^ However, the results are difficult to apply to modern‐day clinical decision making, as patients with metastatic and nonmetastatic disease were often grouped, and patients with A‐SCC histology were often analyzed with the SCC cohorts. Our study shows that patients with A‐SCC undergo surgery at significantly higher rates than do SCC patients (~3‐fold), with better survival outcomes more closely resembling AC than SCC.

Population‐based studies of pancreatic SCC have previously shown more adverse features for pancreatic SCC, including high‐grade histology, and inferior survival.^9^ Our analysis is the largest and the first to examine survival in nonmetastatic disease based on treatments such as definitive surgery, radiation therapy with definitive intent, and chemoradiation therapy. Ours is also the first study to demonstrate advanced stage at presentation for SCC vs AC, possibly leading to lower resection rates. This has prognostic importance, as prior studies show that surgical resection dramatically improves survival.^15^ This is true for pancreatic AC histology,^15^ A‐SCC,^16^ and SCC.^9^ Unfortunately, less than 20% of pancreatic cancer patients have resectable disease at presentation.^17^ Also, prior studies failed to differentiate A‐SCC from SCC, which have a vastly different survival.

We found that patients with nonmetastatic, primary pancreatic SCC were significantly less likely to undergo surgical resection compared to AC patients. This likely reflects the fact that patients with SCC presented with higher tumor stage. It also may reflect the clinician's lack of confidence in the initial diagnoses, as some may suspect the presence of metastatic SCC, as well as the lack of evidence‐based treatment guidelines for patients with primary nonmetastatic pancreatic SCC. Prior studies do not comment on receipt of chemotherapy or radiotherapy,^9^ making it difficult to analyze the effect of various definitive treatment modalities independently.

In our study, median survival in patients with nonmetastatic SCC of the pancreas was threefold higher for patients who underwent surgical resection, compared to those who did not (21.3 months vs 6.8 months). Surgical resection similarly benefited patients with AC, improving MS by approximately twofold (MS 22.1 months vs 10.0 months). Median survival for SCC patients who underwent surgical resection was only slightly lower than MS for AC patients who underwent surgery (21.3 months and 22.1 months, respectively); this illustrates that in SCC patients who are thoroughly evaluated and have upfront resectable disease, survival is relatively high compared with previous estimates. These data highlight the importance of a complete workup at initial diagnosis of pancreatic SCC, to ensure that all surgically resectable patients are identified and subsequently evaluated by a pancreatic surgery specialist. It also argues in favor of neoadjuvant chemotherapy (or chemoradiotherapy) to patients with newly diagnosed, borderline resectable or unresectable pancreatic SCC, to promote downstaging and increase the chance of a successful future resection. In our study, chemotherapy improved survival by ~50% in patients with nonmetastatic AC (MS of 10.5 months vs 15.6 months); however, chemotherapy was not associated with improved survival on UVA for patients with SCC, with an MS of 8.3 months for those receiving chemotherapy and 8.9 months for those not receiving chemotherapy.

When considering these results, the following limitations should be taken into account. First, although we only included cases with histological confirmation of diagnosis (AC, A‐SCC, or SCC), considering the rarity of both A‐SCC and SCC, it is possible that reviewing pathologists could have misclassified pathological specimens in this rare disease class. Second, the decreased resection rate of SCC also makes it more likely that an A‐SCC could have been misclassified as an SCC in those that were only biopsied. Third, as this is a large national database and a chart review was not available for each patient, we were unable to confirm that all patients in our SCC and A‐SCC group had a complete metastatic workup to exclude SCC of nonpancreatic origin (head and neck or anus, for example) which had metastasized to the pancreas, and would likely respond to localized treatment differently than would primary pancreatic SCC. To help minimize the risk of capturing metastatic SCC to the pancreas, and to make the results more applicable to clinical decision making, we excluded all patients with metastatic disease or unknown metastatic disease status. However, the NCDB does not provide information regarding what was involved in the initial workup for each patient, and it is likely that in our cohort of nonmetastatic patients, a significant percentage had occult metastatic disease. It is also likely that we excluded some patients who the NCDB classified as having metastatic disease who in fact had nonmetastatic disease. Fourth, due to the small sample size of SCC cases, we were unable to propensity match, which may have overestimated the prognostic significance of SCC histology, as these patients tended to present with more aggressive clinicopathologic disease. Additionally, given that one of our study groups (AC) has a very large sample size, it is possible to achieve statistical significance even with very small group differences, so attention should be paid to the clinical significance of observed differences between the three groups as presented in Table 1. Finally, the lack of survival benefit for radiation therapy may be due to insufficient power to show a benefit that would be expected to be small if present.

## 5 CONCLUSION

This study represents the first large‐scale aggregate database analysis of patients with nonmetastatic, primary pancreatic SCC and A‐SCC. It illustrates that even in the nonmetastatic setting, SCC of the pancreas presents with the more aggressive clinicopathologic disease, which is less often surgically resected or treated with any definitive local therapy. This may result in an inferior survival. In patients with primary, nonmetastatic SCC of the pancreas, surgical resection provides the most significant survival benefit, with systemic chemotherapy providing a less significant benefit, and localized radiation providing no statistical benefit for any subgroup.

## CONFLICT OF INTEREST

The authors have no conflicts of interest to declare.

## AUTHOR CONTRIBUTIONS

Conceptualization was done by Ignacio Garrido‐Laguna; Data curation was done by Samual Francis, Joshua Gruhl; Methodology was conceived by Shane Lloyd, Randa Tao, Kajsa Affolter; Formal Analysis was done by Joshua Gruhl, Shane Lloyd; Funding was acquired by n/a; Writing—original draft was done by Joshua Gruhl; Writing—review and editing were done by Joshua Gruhl, Samual Francis, Shane Lloyd, Randa Tao, Ignacio Garrido‐Laguna, Kajsa Affolter; Project administration was done by Shane Lloyd.

## Data Availability

The data that support the findings of this study are openly available in the National Cancer Database at https://www.facs.org/quality-programs/cancer/ncdb/publicaccess.
