# Supplementary figures and images for: The impact of squamous cell carcinoma histology on outcomes in nonmetastatic pancreatic cancer
Source: Cancer Med. 2020 Jan 16;9(5):1703–11. doi: 10.1002/cam4.2851 (PMC7050091; doi:10.1002/cam4.2851)

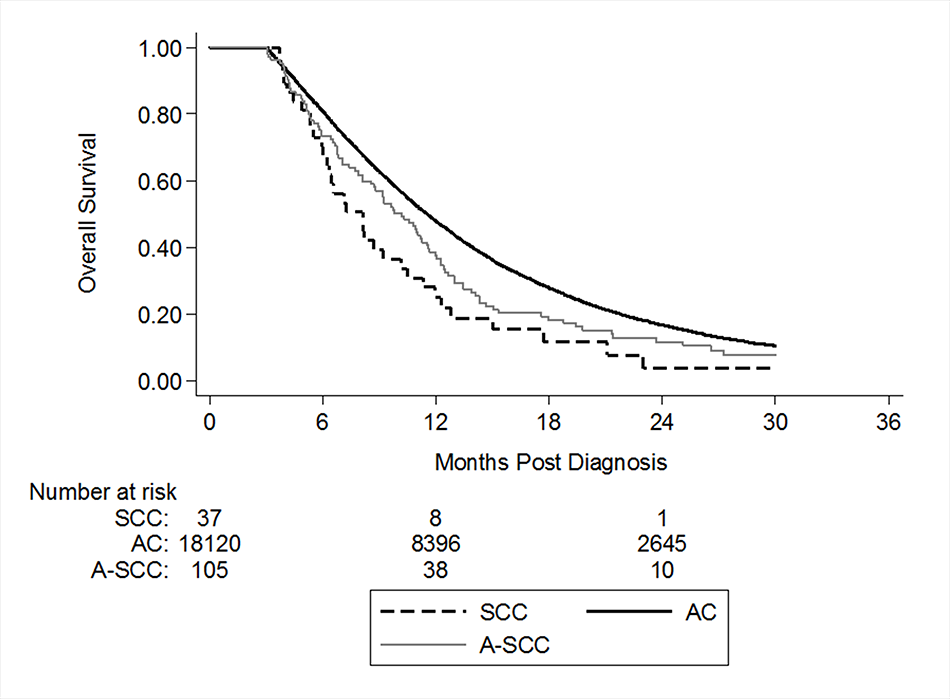

Supplement: Supplementary file 1 [file CAM4-9-1703-s001.tif]
