# Supplementary material for: The impact of squamous cell carcinoma histology on outcomes in nonmetastatic pancreatic cancer
Source: Cancer Med. 2020 Jan 16;9(5):1703–11. doi: 10.1002/cam4.2851 (PMC7050091; doi:10.1002/cam4.2851)
Supplement: Supplementary file 2 [file CAM4-9-1703-s002.docx]

**Supplemental Table 1.** Factors Predictive of Squamous Cell Carcinoma Histology

| **Multivariable Logistic** | **OR** | **95% CI** | | ***P-value*** |
| --- | --- | --- | --- | --- |
| **Age** | 1.04 | 0.98 | 1.10 | 0.20 |
| **Sex** |  |  |  |  |
| Male | Reference |  |  |  |
| Female | 0.63 | 0.2 | 1.95 | 0.42 |
| **Charlson comorbitity score** |  |  |  |  |
| 0 | Reference |  |  |  |
| ≥1 | 0.46 | 0.12 | 1.71 | 0.25 |
| **Tumor Location** |  |  |  |  |
| Head | Reference |  |  |  |
| Body | 4.14 | 1.29 | 23.97 | **0.02** |
| Tail | 4.20 | 1.62 | 23.43 | **0.01** |
| Other | 1.10 | 0.12 | 9.69 | 0.93 |
| **Tumor Grade** |  |  |  |  |
| Low | Reference |  |  |  |
| High | 10.48 | 2.29 | 48.10 | **<.01** |
| **LVSI** |  |  |  |  |
| Negative | Reference |  |  |  |
| Positive | 0.58 | 0.18 | 1.88 | 0.37 |
| **Clinical Stage Group** |  |  |  |  |
| I | Reference |  |  |  |
| II | 1.11 | 0.33 | 3.71 | 0.87 |
| III | 5.02 | 0.61 | 41.60 | 0.14 |
| **Resectability** |  |  |  |  |
| Resectable/Borderline  Resectable | Reference |  |  |  |
| Unresectable | 0.26 | 0.02 | 3.87 | 0.33 |
| **Treated with surgery** |  |  |  |  |
| No | Reference |  |  |  |
| Yes | 0.49 | 0.09 | 2.98 | 0.44 |
| **Treated with radiation (definitive)** | |  |  |  |
| No | Reference |  |  |  |
| Yes | 0.14 | 0.02 | 1.13 | 0.07 |

Abbreviations: OR, odds ratio; CI, confidence interval; LVSI, lymphovascular space invasion.
