# Supplementary material for: The impact of squamous cell carcinoma histology on outcomes in nonmetastatic pancreatic cancer
Source: Cancer Med. 2020 Jan 16;9(5):1703–11. doi: 10.1002/cam4.2851 (PMC7050091; doi:10.1002/cam4.2851)
Supplement: Supplementary file 3 [file CAM4-9-1703-s003.docx]

**Supplemental Table 2.** Cox Analysis for Non-Metastatic Primary Pancreatic Squamous Cell Carcinoma

|  | **UVA** |  |  | **MVA** |  |  |
| --- | --- | --- | --- | --- | --- | --- |
|  | **HR** | **95% CI** | **p-value** | **HR** | **95% CI** | **p-value** |
| **Age** |  |  |  |  |  |  |
| ≤ 50 | ref |  |  | ref |  |  |
| >50 | 0.86 | (0.44, 1.70) | 0.67 | 0.73 | (0.12, 4.61) | 0.74 |
| **Sex** |  |  |  |  |  |  |
| Male | ref |  |  | ref |  |  |
| Female | 1.31 | (0.92, 1.85) | 0.13 | 2.11 | (0.92, 4.84) | 0.08 |
| **Charlson Comorbidity Score** |  |  |  |  |  |  |
| 0 | ref |  |  | ref |  |  |
| 1 | 1.06 | (0.72, 1.57) | 0.77 | 1.64 | (0.60, 4.49) | 0.33 |
| 2+ | 0.94 | (0.47, 1.86) | 0.85 | 0.56 | (0.13, 2.40) | 0.44 |
| **Grade** |  |  |  |  |  |  |
| Low/Intermediate | ref |  |  | ref |  |  |
| High | 2.54 | (1.28, 5.05) | **0.01** | 1.58 | (0.54, 4.61) | 0.41 |
| **Clinical T-stage** |  |  |  |  |  |  |
| cT1 | ref |  |  | ref |  |  |
| cT2 | 2.62 | (0.76, 8.99) | 0.13 | 8.88 | (1.57, 50.17) | **0.01** |
| cT3 | 3.18 | (0.99, 10.26) | **0.05** | 15.13 | (2.75, 83.17) | **0.00** |
| cT4 | 4.05 | (1.24, 13.22) | **0.02** | 6.03 | (1.02, 35.59) | **0.05** |
| **Clinical N-stage** |  |  |  |  |  |  |
| cN0 | ref |  |  | ref |  |  |
| cN1 | 1.09 | (0.71, 1.69) | 0.69 | 0.85 | (0.36, 2.02) | 0.72 |
| **Treated with Surgery** |  |  |  |  |  |  |
| No | ref |  |  | ref |  |  |
| Yes | 0.22 | (0.13, 0.36) | **0.00** | 0.19 | (0.07, 0.54) | **0.00** |
| **Treated with Radiation** |  |  |  |  |  |  |
| No | ref |  |  | ref |  |  |
| Yes | 1.48 | (0.96, 2.26) | 0.08 | 1.10 | (0.39, 3.11) | 0.86 |
| **Treated with Chemotherapy** |  |  |  |  |  |  |
| No | ref |  |  | ref |  |  |
| Yes | 1.13 | (0.77, 1.64) | 0.61 | 0.22 | (0.08, 0.63) | **0.01** |

Abbreviations: UVA, univariate analysis; MVA, multivariate analysis; HR, hazard ratio; CI, confidence interval.
