# Supplementary material for: The impact of squamous cell carcinoma histology on outcomes in nonmetastatic pancreatic cancer
Source: Cancer Med. 2020 Jan 16;9(5):1703–11. doi: 10.1002/cam4.2851 (PMC7050091; doi:10.1002/cam4.2851)
Supplement: Supplementary file 4 [file CAM4-9-1703-s004.docx]

**Supplemental Table 3. Cox analysis for non-metastatic primary pancreatic SCC.**

|  |  | UVA | | | | MVA | | | |
| --- | --- | --- | --- | --- | --- | --- | --- | --- | --- |
|  |  | HR | 95% CI | | p-value | HR | 95% CI | | p-value |
| **Age** |  |  |  |  |  |  |  |  |  |
| ≤ 50 |  | 1.00 | 1.00 | 1.00 |  | 1.00 | 1.00 | 1.00 |  |
| >50 |  | 0.86 | 0.44 | 1.70 | 0.67 | 0.73 | 0.12 | 4.61 | 0.74 |
| **Sex** |  |  |  |  |  |  |  |  |  |
| Male |  | 1.00 | 1.00 | 1.00 |  | 1.00 | 1.00 | 1.00 |  |
| Female | | 1.31 | 0.92 | 1.85 | 0.13 | 2.11 | 0.92 | 4.84 | 0.08 |
| **Charlson Comorbidity Score** | | |  |  |  |  |  |  |  |
| 0 |  | 1.00 | 1.00 | 1.00 |  | 1.00 | 1.00 | 1.00 |  |
| 1 |  | 1.06 | 0.72 | 1.57 | 0.77 | 1.64 | 0.60 | 4.49 | 0.33 |
| 2+ |  | 0.94 | 0.47 | 1.86 | 0.85 | 0.56 | 0.13 | 2.40 | 0.44 |
| **Grade** |  |  |  |  |  |  |  |  |  |
| Low/Intermediate | | 1.00 | 1.00 | 1.00 |  | 1.00 | 1.00 | 1.00 |  |
| High |  | 2.54 | 1.28 | 5.05 | **0.01** | 1.58 | 0.54 | 4.61 | 0.41 |
| **Clinical T-stage** | |  |  |  |  |  |  |  |  |
| cT1 |  | 1.00 | 1.00 | 1.00 |  | 1.00 | 1.00 | 1.00 |  |
| cT2 |  | 2.62 | 0.76 | 8.99 | 0.13 | 8.88 | 1.57 | 50.17 | **0.01** |
| cT3 |  | 3.18 | 0.99 | 10.26 | **0.05** | 15.13 | 2.75 | 83.17 | **0.00** |
| cT4 |  | 4.05 | 1.24 | 13.22 | **0.02** | 6.03 | 1.02 | 35.59 | **0.05** |
| **Clinical N-stage** | |  |  |  |  |  |  |  |  |
| cN0 |  | 1.00 | 1.00 | 1.00 |  | 1.00 | 1.00 | 1.00 |  |
| cN1 |  | 1.09 | 0.71 | 1.69 | 0.69 | 0.85 | 0.36 | 2.02 | 0.72 |
| **Treated w/ Surgery** | |  |  |  |  |  |  |  |  |
| No |  | 1.00 | 1.00 | 1.00 |  | 1.00 | 1.00 | 1.00 |  |
| Yes |  | 0.22 | 0.13 | 0.36 | **0.00** | 0.19 | 0.07 | 0.54 | **0.00** |
| **Treated w/ Radiation** | |  |  |  |  |  |  |  |  |
| No |  | 1.00 | 1.00 | 1.00 |  | 1.00 | 1.00 | 1.00 |  |
| Yes |  | 1.48 | 0.96 | 2.26 | 0.08 | 1.10 | 0.39 | 3.11 | 0.86 |
| **Treated w/ Chemotherapy** | |  |  |  |  |  |  |  |  |
| No |  | 1.00 | 1.00 | 1.00 |  | 1.00 | 1.00 | 1.00 |  |
| Yes |  | 1.13 | 0.77 | 1.64 | 0.61 | 0.22 | 0.08 | 0.63 | **0.01** |
