# Supplementary material for: The impact of squamous cell carcinoma histology on outcomes in nonmetastatic pancreatic cancer
Source: Cancer Med. 2020 Jan 16;9(5):1703–11. doi: 10.1002/cam4.2851 (PMC7050091; doi:10.1002/cam4.2851)
Supplement: Supplementary file 5 [file CAM4-9-1703-s005.docx]

**Supplemental Table 4.** Reported Cases of Primary Squamous Cell Carcinoma of the Pancreas in the Literature

| **Author** | **Year** | **Institution** | **Study Design** | **No. of patients** | **Median Age (years)** | **Median Survival** | **% of patients with metastatic disease** | **Treatment Received** | **Notable Findings** |
| --- | --- | --- | --- | --- | --- | --- | --- | --- | --- |
| Makarova-Rusher, O | 2016 | NCI | SEER Database Review | 214 | 68 years | 4 months (10 months in surgical cohort; 4 months in non-surgical cohort). | 92/214 pts (43.0%) | Surgery: 22/214 (10.3%) |  |
| Ntanasis-Stathopoulos, I | 2016 | Alexandra Hospital (Greece) | All cases from literature review | 54 | 62 years | 7 months | 28/51 pts (54.9%) | Surgery: 17/54 (31.5%); CRT: 8/34 (23.5%); CHT alone: 16/34 (47.1%); RT alone: 6/34 (17.6%) | Resectability (p=.003) and more recent publication year (p<.001) associated with better OS |
| Brown, HA | 2005 | Yale | 1 case from institution; 24 cases from literature review | 25 | 62 years | 7 months (range, 6-16 months) if undergoing curative resection; 3 months if no curative resection (range, 0.25-9 months) | Not reported | Surgery: 7/25 (28%); CHT alone: 1/25; CRT: 5/25; RT alone: 4/25 |  |
| De Souza, AL | 2014 | Tufts | All cases from literature review | 10 | 66 years | 9 months (range, 1.5 - 15 months) | 6/10 pts (60.0%) | Surgery: 2/10 (20%); CHT alone: 4/10 (40%); RT alone: 1/10 | Longest survival 15.0 months after treatment with cisplatin and 5-FU |
| Mansfield, A | 2010 | Mayo Clinic | All cases from Mayo Clinic Experience | 8 | 64 years | 5 months | 5/8 pts (62.5%) | Surgery alone: 1/10; Surgery +RT: 1/10; Surgery/RT/CHT: 3; CHT alone: 1/10 |  |
| Zhang, G | 2018 | Sichuan Cancer Hospital | All cases from literature review | 6 | 71 years | 2.5 months (range, 1-4 months) | 4/6 pts (66.7%) | CHT alone: 3/6; CRT: 1/6; RT alone: 1/6 |  |

Abbreviations: No., number; NCI, National Cancer Institute; SEER, Surveillance, Epidemiology, and End Results; pts, patients; CRT, chemoradiotherapy; CHT chemotherapy; RT, radiation therapy; OS, overall survival.
